# Supplementary material for: Mentors matter: Association of mentors with project success in the Apache Software Foundation Incubator
Source: PLoS One. 2022 Aug 17;17(8):e0272764. doi: 10.1371/journal.pone.0272764 (PMC9384984; doi:10.1371/journal.pone.0272764)
Supplement: Attachment — Submitted filename: Mentors Matter - response to reviewers.docx [file pone.0272764.s003.docx]

Editor Naldi,

Thank you for your handling of this paper. The reviewers you solicited provided great thoughts. Incorporating their comments has made the paper stronger. I respond here to the comments from reviewers. Hopefully this allows the paper to be published. Please let me know if you have any questions or comments.

Reviewer 1:

Lines 37, 39-40, 44, 50-53, 96, 104-107, 198-199 were dealt with as suggested by the reviewer

Line 376-377: The statement was clarified as just qualifying our results. As stressed above, we are not testing whether having a mentor matters, but whether specific mentors matter

1)

This is addressed in the revision on lines 277-278

2)

This is addressed in the revision on lines 203-206

3)

Unfortunately, these data do not exist in this dataset. This is a good avenue for further research. This had been added into the new “Threats to validity” subsection”

4)

This is shown in Figure 3 and addressed in the paragraph spanning from lines 296-308. A simple correlational test does show that there is a relationship, though (as noted in the prose), that simple result hides some important variation in the distribution of graduation rate by number of projects mentored. I believe that presenting the simple correlation would obfuscate the important underlying dynamics.

5)

This was previously address in the “Implications for future research”. This has been expanded slightly with a specific idea to investigate what makes for (un)successful mentoring (lines 426-432).

There have been some insinuations of threats to validity (such as pointing out selection bias in mentors choosing projects likely to graduate) but these threats to validity should be explicitly stated. It warrants having a dedicated (sub)section.

A threats to validity subsection was created.

The figures are out of order, but I will assume that will be fixed for the camera-ready version.

I believe that I submitted the Figures correctly labeled. Please let me know if I have erred. Thanks!

Figure 1 should have more ticks (with labels) because it is hard to interpret whether each bar is "bucketing" more than one number or if there is something else going on

 Ticks have been added for each bucket

Figure 4 is the y-axis rate (i.e. rate for number of mentors is better than chance) or percentage (as labelled, which means <1% graduation rate for most). I suspect the former, but if that is the case, the axis label should be fixed.

The y-axis is % of projects with that many mentors that have graduated. Fig title has been changed to be consistent and axis label has been expanded

Reviewer 2:

In the introduction section, I suggest that the authors add a new paragraph addressing the research gap, introduce the focal research question, and add a paragraph presenting the manuscript structure/content.

Added this paragraph lines 82-91

- In the introduction, the acronym “ASF” (Apache Software Foundation) should be defined before the first use.

Corrected
- The reference list should be revised since some references are incomplete (ex. ref. 22).

Corrected

Thank you for your time!

Curtis Atkisson
