# Supplementary material for: Mentors matter: Association of mentors with project success in the Apache Software Foundation Incubator
Source: PLoS One. 2022 Aug 17;17(8):e0272764. doi: 10.1371/journal.pone.0272764 (PMC9384984; doi:10.1371/journal.pone.0272764)
Supplement: Attachment — Submitted filename: Mentors Matter - response - 2.docx [file pone.0272764.s004.docx]

Editor Naldi,

Thank you for your handling of this paper. I have incorporated the suggestions of Reviewer 1 and took the opportunity allowed by Reviewer 2 to expand on the Discussion. Please let me know if you have any questions or comments.

1. The representation of Figure 4 is still incongruent with the other data presented. The y-axis suggests that the percentage of projects that graduated ranges from 0.5% to 1% (depending on number of mentors) and that the vast majority of projects are not represented in this chart. I suspect this is a problem of the scale label, where 0.5% should really be 50% and 1.0% should really be 100%. If that is the case, the scale should be fixed (or axis label corrected to clarify that it is the graduation *rate*, and not the *percentage*).

THIS HAS BEEN CORRECTED

2. Even with more tick marks, Figure 1 is still difficult to interpret. The ticks fall in between (and not aligned with) each bar. Consequently, it is unclear at x=2 if the y-value is ~175 or ~50.

INSTEAD OF HAVING TICK MARKS FOR EACH INTEGER, THE FIGURE NOW HAS TICK MARKS ONLY FOR EACH BIN IN THE HISTOGRAM. EACH BIN SPANS THE TICK MARKS AROUND IT. TO MY EYE, THIS IS A STANDARD WAY OF PRESENTING A HISTOGRAM.

3. Lines 404-5 state: "Ongoing qualitative work with mentors, however, indicates that is not the case." There is no substantiation provided. If there are publications of this work, please cite them; if there are novel data, please present them (along with the methods). Otherwise, it should be restated that the authors believe it is not the case based on their anecdotal observations.

THIS HAS BEEN RESTATED AS ANECDOTAL EVIDENCE. THE QUALITATIVE WORK IS CURRENTLY BEING WRITTEN UP FOR PUBLICATION, SO HAS NO FORMAL CITATION AS YET

Reviewer #2: I am happy with the revision made by the author.
As I said in my previous review, the manuscript would benefit from a more elaborate discussion, comparing the obtained results with the prior literature. The author did not make this improvement, but the manuscript quality is sufficient for publication.

I HAVE INCLUDED AN EXPANDED DISCUSSION OF THE IMPACTS THIS FINDING SHOULD HAVE ON THE STUDY OF OSS AND MENTORING IN THE MAIN BODY OF THE DISCUSSION SECTION, BEFORE THREATS TO VALIDITY

Thank you for your time!

Curtis Atkisson
